# The impact of an aging population on the Portuguese healthcare system—the primary healthcare perspective and the potential of the WHO ICOPE approach

**DOI:** 10.3389/fpubh.2026.1942276

**Published:** 2026-09-25

**Authors:** António Luís Vidinha Pereira, Constança Paúl, Ana Vila Flor, Sara Castendo Ramos, Inês Rosendo, Luiz Santiago

**Affiliations:** 1 ULS Coimbra – Unidade Local de saúde de Coimbra, Coimbra, Portugal; 2 ICBAS - Instituto de Ciências Biomédicas Abel Salazar, Universidade do Porto, Porto, Portugal; 3 CINTESIS - Center for Health Technology and Services Research, Faculty of Medicine, University of Porto, Porto, Portugal; 4 Faculty of Medicine, University of Coimbra, Coimbra, Portugal; 5 General Practice/Family Medicine Clinic, University of Coimbra, Coimbra, Portugal; 6 Centre of Health Studies and Investigation of the University of Coimbra, Coimbra, Portugal

**Keywords:** aging, comprehensive geriatric assessment, integrated Care for Older People, Portugal, primary healthcare

## Abstract

Portugal is one of the countries where the challenges associated with an aging population are most significant. In this context, a reformulation of public health policies is required, specifically, the urgent adaptation of the Portuguese National Health Service (SNS). Founded in 1979, when Portugal had one of the youngest populations in Europe, the SNS was primarily oriented toward improving maternal-child health indicators, with primary healthcare (PHC) playing a key role. At present, the SNS needs to adapt, as some data suggest it is struggling to keep pace with the aging trend. The present SNS approach to geriatric care highlights chronic disease, multimorbidity, loss of capacity, frailty, and disability, portraying older adults as a collection of deficits requiring formal treatment. The SNS lacks professionals with solid geriatric training working in the community and forming multidisciplinary teams, with proper pathways connecting community resources to hospitals. Several studies worldwide demonstrate the adaptability, feasibility, effectiveness and scalability of the WHO ICOPE approach. However, concerns have been raised regarding its implementation, especially in low- and middle-income countries, mainly due to time constraints and lack of human and financial resources. Our research team is developing pilot fieldwork using ICOPE to better understand the capabilities of this framework. By presenting this perspective, we aim to contribute to the discussion on the potential of ICOPE within the specific Portuguese demographic context, including eventual challenges and facilitators to its adaptation in PHC, and its potential contribution to the integration of care across levels in the Portuguese setting.

## Introduction

The concept of population aging—the proportional increase of older adults within the population—is relatively new. In 1950, no country had more than 11% of its population aged 65 and over. In 2000, the highest proportion was 18% (1). However, worldwide projections for 2050 indicate that, for the first time, there will be a larger number of older adults than adolescents (2.1 billion versus 2.0 billion) (2). Regarding people aged 80 years or older, the numbers grow even faster (3), reflecting the increase in average life expectancy with improvements in public health. This should be seen as an achievement rather than a problem (1).

The aging process is more advanced in some regions. The proportion of older adults tends to be higher in Europe, while absolute numbers are higher in Asia (4). Japan has the world’s most aging population (30%), followed closely by Finland, Italy, Spain and Portugal (5). Given this context, our aim is to examine the Portuguese situation. Portugal is a western European country with part of its territory on the mainland and the other spread across two Atlantic archipelagos (Azores and Madeira autonomous regions). The population is 10.7 million, and 21.8% is aged 65 or over, while the European Union (EU) average is 20.0% (6). In 2080, Portugal’s population is expected to decline to 7.5 million. The number of older adults will rise from 2.1 to 2.8 million. The aging index will almost double, from 147 to 317 older adults per 100 younger people (7). There is also a shift of the interior population to more attractive coastal cities, causing asymmetry in population distribution (8, 9). This situation is not expected to be significantly altered by immigration (7).

These changes can impact society and must be addressed proactively (3). More important than focusing on reversing demographic trends, it is imperative to secure the future of older adults with effective approaches (10). A crucial area that needs reshaping is long-term healthcare policy, more specifically, the urgent adaptation of the Portuguese National Health Service (SNS) to the new demographic reality (11, 12).

The SNS was launched in 1979 in a different demographic setting from the current one, and it succeeded in addressing the public health priorities of the time. In the 1970s, Portugal had one of the youngest populations in Europe (10). It also had the worst maternal-child health indicators (13). To change the way healthcare was provided, Portugal drew on best practices and adapted the cornerstones of the British National Health Service (NHS) to establish the SNS (14), a fully integrated system conceived from a public health perspective - university training, professional placement within the system, patient entry via primary healthcare (PHC), integration with hospitals, use of the social-private sectors when the public system required it (15). Health indicators improved swiftly, particularly those related to maternal–infant mortality, rapidly placing Portugal among the world’s most developed countries in this field. In 1980, the infant mortality rate (per 1,000 live births) was 14.6 in the EU and 24.3 in Portugal. In 2003, it was 4.6 in the EU and 4.0 in Portugal (15).

However, Portugal’s needs are very different today, and it is essential that the SNS adapts to this new demographic reality (16, 17). Average life expectancy in Portugal has risen significantly, from 67.7 years for men and 75.2 years for women in 1980 to the current 78.73 years for men and 83.96 years for women. In the EU, average life expectancy is now 78.9 years for men and 84.1 years for women (18). Also, some data suggest the SNS is struggling to keep pace with the aging trend. Portuguese women have only 7.8 healthy-life years after the age of 65, compared with the EU average of 9.6 years and 14.3 years in Sweden (19). Evidence suggests a considerable burden of health conditions among Portuguese older adults, particularly chronic non-communicable diseases (20, 21), with incidence probably influenced by factors such as social conditions (15), poor diets, harmful habits (such as alcohol consumption and smoking), sedentary lifestyle (20) as well as isolation, depression and anxiety (21), all hindering healthy aging. For example, Portugal is one of the EU countries where the fewest people take part in sport or physical activity of any form (15). Portugal has one of the highest prevalences of hypertension in Europe (3 in 10 individuals) and around 100 Portuguese die every day from cerebro-cardiovascular diseases (21). Around 41% of healthy-life years lost in Portugal could have been prevented if the main modifiable risk factors had been eliminated (21).

This difficult situation persists despite substantial investment. Portugal’s current healthcare expenditure stands at around 10.2% of GDP, equivalent to €2,733 per capita. By 2050, a rise to €3,834 is expected, with public investment growing by 28.6% (21). However, households bear 29 to 38% of total costs through direct payments (co-payments, consultations and medicines), among the highest in the OECD (22). Older adults have the highest direct healthcare costs. In 2015, while the national average for direct healthcare expenditure was €445.26 per capita, older adults spent €692.00, with the highest amount among those aged 85 or over: €880.20 (22).

Beyond considerations of healthcare investment, attention should also be given to how care is organized and delivered. In this context, the geriatric care pathway, originally designed in 1979, warrants consideration as a potential contributor to some of the challenges observed today (15, 23). Although PHC is the gateway to the SNS (14), geriatric care is centered mainly on Internal Medicine (IM) (15). Some authors consider this a reason why caring for older adults in Portugal can lead to reactive interactions within PHC and hospitals, probably resulting in frequent visits to emergency departments (15, 24). This framing highlights chronic disease, multimorbidity, loss of capacity, frailty and disability, portraying older adults as a collection of deficits in need of formal treatment (24). Furthermore, Geriatrics is not a standalone speciality in Portugal. Doctors interested in this field may apply to the Portuguese Medical Association (OM) for an assessment of their CV to be certified as “competent in Geriatrics.” However, only 138 doctors currently hold this qualification (25). Consequently, the shortage of training in this field may hinder the formation of teams, the referral of complex cases and clinical supervision (26). Therefore, Portugal could benefit from professionals with solid geriatric training, who can understand person-centered care, the preferences and goals of older adults, autonomy, social participation, and the relationship between individual characteristics, context and community resources (11, 27, 28). Several authors consider that PHC professionals should be included in this group, establishing effective communication with IM and integrating levels of care (15, 20, 24, 29, 30). Firstly, there is the issue of proximity. Health-centers—where Portuguese PHC professionals carry out their work—are spread across all municipalities and play a key role, particularly in rural areas (15). With multidisciplinary teams attentive to the top of the age pyramid, PHC could focus on prevention and early diagnosis, establishing well-planned, person-centered, holistic approaches for older adults (30). Addressing short-, medium- and long-term community care objectives in this field could improve the well-being of older adults and their families (20, 30). If hospital-based responses or other medical specialities are required in a more complex situation, these should be provided through proper pathways, not simply involving visits to emergency departments not tailored to older adults’ needs (24).

## Discussion—finding ways of adapting Portuguese PHC to an aging population

Our research group has been working to identify, adapt and develop high-quality and cost-effective ways of integrating Portuguese PHC into care for older adults (31–33). Given the structure of the SNS, we realized that, to liaise with IM (the speciality leading geriatric care), we needed to understand their approach.

IM mostly uses a multimorbidity and frailty-based approach embodied by the comprehensive geriatric assessment (CGA) (29, 34), a multidimensional, multidisciplinary assessment of physical health, cognition, mental health, and social circumstances. The CGA aids in the diagnosis of medical conditions, development of treatment and follow-up plans, coordination of care, evaluation of long-term care needs and effectiveness of instituted therapeutics (35). Validated tools and survey instruments are an important part of CGA (35), and they have given rise to concise, quantifiable, and measurable models, understood by clinicians, health managers, and policy-makers (36).

In our first study (33), we drew on the framework provided by the Centre for Geriatric Studies of the Portuguese Society of Internal Medicine (GERMI), suggesting assessment tools validated for Portugal, namely, the Katz Scale, Lawton-Brody Scale, Holden’s Functional Gait Classification, Yesavage Geriatric Depression Scale (short version), Mini-Mental State Examination, and the Mini-Nutritional Assessment (37). Like other authors, we concluded that this form of CGA can be well suited to hospital setting; however, it rarely adapts to community-dwelling individuals (33, 38). As future courses of action, we highlighted the need for easier and quicker tools to use in PHC, integration of these tools into electronic health records to increase monitoring and feedback, adjustment of consultation times to suit appropriate assessment of older adults, training of medical students, doctors and other professionals (such as nurses and social workers) in the use of such tools, and development of protocols for clinical and organizational coordination between PHC and hospitals (33). Above all, we realized that PHC needed to look at older adults as a whole, focusing beyond frailty and disability to support aging in place, including nonmedical domains, biopsychosocial factors, social networks, older adults’ coping strategies, and emphasizing functional capacity and quality of life (33).

Consequently, we found brief screening instruments developed worldwide to promote a more geriatric-responsive culture in PHC, for example, the Rapid Geriatric Assessment (39); Gerontopôle Screening Tool (40); Kihon Checklist (KCL) (41); Vulnerable Elders 13 Survey (VES-13) (42); or the Easycare Two-step Older Persons Screening (Easycare-TOS) (43). However, some authors indicate that these instruments have difficulty becoming firmly established (44).

Another limitation is that in some industrialized countries, outpatient CGA is performed by specialized geriatricians or gerontologists instead of the Portuguese PHC professionals (doctors, nurses and social-workers) (31, 45). A recent systematic review including 31 studies found considerable variation in team composition for outpatient CGA, as PHC doctors were only involved in 12 studies (46), suggesting limited and insufficient approaches to implementing CGA in the Portuguese PHC setting. There was also great heterogeneity in assessment instruments and care coordination models, possibly reflecting contextual adaptations and structural challenges (46).

Moving forward, we planned a literature review (31) to compile and compare CGA models worldwide and their applicability by PHC professionals. The study attracted some interest, demonstrating that this is a topical and relevant issue (47, 48). One study building on our work identifies 16 structured clinical tools to assist clinicians in PHC (47), including some of those mentioned above and others, for example, Flaherty’s Health Maintenance Clinical Glidepaths, Hong Kong Reference Framework, WHO ICOPE (Integrated Care for Older People) or the Manageable Geriatric Assessment (MAGIC) (47). The authors conclude that no structured model currently meets the practical and clinical expectations of PHC professionals; however, they note that an ideal model would offer a structured framework guiding patient-centered care while still allowing room for clinician judgment, respecting individual needs and acknowledging both resilience and vulnerability (47). Another study citing our work states that emerging concepts such as intrinsic capacity (IC) and functional ability (FA) are important in addressing limitations of traditional models, and considers the classical care model based only on disease management insufficient (48). IC is the composite of all physical and mental capacities an individual can draw. FA is the health-related attribute that enables people to be and to do what they have reasons to value (49).

Building on the growing recognition of these concepts, our understanding of the needs of the SNS, and the recommendations outlined in the WHO’s 2015 World Report on Aging and Health (50), we developed a strong interest in the WHO ICOPE approach. Although as noted earlier, no framework provides an ideal solution for all contexts, we consider that ICOPE has the potential to support greater integration across levels of the SNS while allowing for clinical judgment and taking into account older adults’ preferences, wishes, and individual circumstances. ICOPE recognizes that the onset or progression of frailty and care dependency may be delayed, slowed, or, in some cases, partially reversed through interventions initiated early in the process of functional decline and declining intrinsic capacity (49). To operationalize this approach, ICOPE encompasses several pathways addressing physical, mental, and social dimensions of health, with attention to the circumstances, preferences, and wishes of each individual. When a potential decline in intrinsic capacity or functional ability is identified, a more comprehensive assessment can then be undertaken, informing the development of an integrated and holistic care plan that may also incorporate social and caregiver support (51). Importantly, this approach does not replace specialized care when required; rather, hospital, geriatric, and other specialized services remain available for referral when more complex care needs arise (44, 51).

Approaching older adults through this lens could be interesting for Portuguese PHC. This hypothesis needs to be substantiated in subsequent structured studies addressing this issue, for example, through a theory of change, as some authors have done for other countries (52, 53). However, we can make a few preliminary observations. Firstly, ICOPE was designed to establish global forms of action in PHC, having the potential to correspond to Portuguese requirements. Furthermore, ICOPE aims to draw attention to problems often overlooked by professionals lacking training in identifying and managing some impairments in older adults, meeting the SNS need for this type of care. Moreover, it is designed for use by various types of PHC professionals—not only doctors, but also nurses and social-workers who can be found in any Portuguese health-center. Furthermore, as PHC is a well-established concept with solid teams already in place, it makes sense to first use these assets, as in other contexts (54, 55), avoiding investments in uncertain resources without knowing whether the concept is applicable in Portugal. Additionally, ICOPE’s priority in integrating care has the potential, through its care pathways, to optimize the link between PHC and hospitals via dedicated channels. This takes on greater significance within the current legal structure of the SNS, with the recent establishment of local health units (ULS) where PHC and hospitals fall under the same management but are still seeking a framework capable of integrating both levels of care (56). The establishment of “Integrated Clinical Pathways” or protocols relating to older adults could, eventually, help optimize and restructure care in this field. Finally, having extensive expertise and capacity in successfully adapting WHO’s recommendations for the national context in other areas (15, 23), Portugal’s know-how could facilitate the assimilation of ICOPE’s guidelines, whose documents are already intended for use by health policymakers and professionals worldwide.

Introduced in 2017, by 2022, the ICOPE framework included 18 study teams and sites across the world (57). In 2026, some authors identified 181 studies across 26 countries, with 88.4% reporting beneficial effects, predominantly among older adults (58). For example, in Sweden, interventions frequently improved subjective health (24%), disability (48%), and service outcomes (58%) (59). In France, the results of the first large-scale pragmatic implementation of ICOPE with more than 10,000 individuals sparked the government’s interest in rolling out the program nationwide (60–62). Growing evidence from the field demonstrates the adaptability (52, 53, 57), feasibility (63), effectiveness (64), and scalability (61, 65) of ICOPE across a wide range of contexts.

However, concerns have also been raised regarding the implementation of ICOPE. A French study revealed that only 12% (56 out of 449) of professionals had completed ICOPE training, with obstacles including time constraints, lack of funding and difficulties in balancing prevention with treatment activities in clinical practice (66). A Swedish study also reports limited and inconsistent outcomes at provider, system, and implementation levels (58). The lack of human and financial resources is a cross-cutting barrier to ICOPE implementation, particularly in low- and middle-income countries (52, 53, 67) where infrastructure barriers are more acute (67). In addition, problems intrinsic to ICOPE have also been described, since the initial ICOPE screening tool exhibited heterogeneous psychometric properties requiring local adaptations to ensure accuracy (68, 69). The most striking shortcoming is the disconnect between screening and intervention, with limited evidence of long-term effectiveness (most studies focus on the initial screening stage and not on the clinical outcomes of subsequent stages) (57).

All these issues may be expected when implementing ICOPE in Portugal, along with others that may arise due to the specific circumstances of our country. Given disparities in population and resources, we can surmise that, particularly in inland areas and on the islands, it may be more difficult for ICOPE to reach its full potential. Despite the widespread national distribution of health-centers, the same cannot be said about some professionals, with Portugal facing a shortage of PHC doctors (70, 71). The same applies to central hospitals (72) and some specialist departments (8, 9, 28), where older adults are referred within the integrated care system. Identifying needs with screening will only have positive clinical outcomes if there is capacity to respond at the next level. Fieldwork will be required in a variety of Portuguese contexts and locations to gain a sound understanding of the potential benefits of ICOPE and challenges involved in implementation.

The latest evidence published worldwide acknowledges that implementation often begins with small-scale pilot projects that test strategies and adapt the model to local contexts (73). We are presently carrying out pilot fieldwork to understand methods and challenges of implementation in Portugal. To the best of our knowledge, our study is currently the only one of its kind being conducted in the country. By presenting this perspective, we aim to assess the current SNS situation and contribute to the discussion on the potential of ICOPE within the specific Portuguese demographic context, broadening knowledge and supporting PHC professionals in achieving better outcomes. Given the limited evidence in Portugal, expanding research across diverse contexts will be essential to identify implementation barriers, assess cost-effectiveness, and inform future investment and eventual implementation.

## Data Availability

The original contributions presented in the study are included in the article/supplementary material, further inquiries can be directed to the corresponding author.
